# Detecting driver stress and hazard anticipation using real‐time cardiac measurement: A simulator study

**DOI:** 10.1002/brb3.2424

**Published:** 2022-01-28

**Authors:** Laora Kerautret, Stephanie Dabic, Jordan Navarro

**Affiliations:** ^1^ Laboratoire d'Etude des Mecanismes Cognitifs (EA 3082) University Lyon 2 Bron France; ^2^ Valeo Interior Controls Rue Jules Verne Annemasse France; ^3^ Institut Universitaire de France Paris France

**Keywords:** anticipation, detection, driver, heart rate change, stress

## Abstract

**Objectives:**

In the context of growing interest in real‐time driver stress detection systems, we question the value of using heart rate change over short time periods to detect driver stress and hazard anticipation.

**Methods:**

To this end, we explored changes in heart rate and speed as well as perceived stress in 27 drivers in a driving simulator. Driver stress was triggered by using hazardous road events, while hazard anticipation was manipulated using three levels of hazard predictability: unpredictable (U), predictable (P), and predictable and familiar (PF).

**Results:**

The main results indicate that using heart rate change (1) is a good indicator for detecting driver stress in real time, (2) provides a cardiac signature of hazard anticipation, and (3) was affected by perceived stress groups. Further investigation is needed to validate the lack of relationship between increased anticipation/predictability and strengthened cardiac signature.

**Conclusions:**

These results support the use of heart rate change as an indicator of real‐time driver stress and hazard anticipation.

## 1 INTRODUCTION

### 1.1 Detecting stress and hazard anticipation: Interest for safety

Drivers are frequently exposed to a variety of hazardous situations that can cause states of stress. Therefore, gaining insight into stress states and stressful situations is of major interest in improving road safety. Several studies have tackled these issues using driving simulators (Napoletano & Rossi, 2018; Paredes et al., 2018; Rendon Velez et al, 2016; Zontone et al., 2021). Although the use of driving simulators is a controversial topic, particularly because it sometimes lacks realism (Jeihani et al., 2020), previous research has proven the ability of artificial environments to detect driver psychological constructs (e.g., stress, anxiety, Tozman et al., 2015; fatigue and drowsiness, Murugan et al., 2020), and to facilitate the understanding of driver behavior in emergency situations (Banerjee et al., 2020). In addition, various driving simulator studies have been able to identify several stressful driving situations (e.g., poor light conditions, Balters et al., 2018; Rigas et al., 2011; bad weather conditions, Funke et al., 2007; complex driving environments and traffic conditions, Rastgoo et al., 2019), also observed in on‐road studies (Healey & Picard, 2005; Rodrigues et al., 2015; Tavakoli et al., 2020) and reported by drivers in large‐scale surveys (Hill & Boyle, 2007).

When a stressful driving situation occurs, the ability to anticipate potential road hazards and traffic situations (i.e., a skill known as hazard perception; Moran et al., 2019) is essential to maximize decision‐making time (Jackson et al., 2009), and thus avoid increased stress levels caused by time urgency (Wickens et al., 2015). One of the main safety challenges is therefore to design in‐vehicle systems able to detect in real time stress states and failures in hazard perception. In addition, these systems would provide alerts to the driver when a lack of hazard anticipation is detected. It is well established that such systems need to rely on real‐time physiological indicators (Rastgoo et al., 2018) in order to be continuously informed about the psychological and physiological states of individuals (Hancock & Warm, 1989).

### 1.2 Toward a real‐time detection of stress and hazard anticipation

In the past, driver stress has been identified based on a wide variety of physiological indicators (Antoun et al., 2017; Rastgoo et al., 2018). Cardiac indicators, including heart rate and heart rate variability, have been by far the most used (Antoun et al., 2018; Gotardi et al., 2019; Haouij et al., 2018; Heikoop et al., 2019; Khattak et al., 2018; Meesit et al., 2020; Reimer et al., 2016). One reason for this preference is related to the ability of cardiac measurements to directly reflect the autonomic nervous system activity (Sztajzel, 2004) underlying the physiological manifestation of stress (Lanatà et al., 2014). Most studies examining driver stress have analyzed cardiac signals over several minutes before averaging them to estimate an overall stress state. Although this method provides good stress detection (Healey & Picard, 2005), it does not permit real‐time detection. Given the growing interest in detecting driver stress more quickly, we question the value of using heart rate change to detect driver stress and hazard anticipation over short time periods.

Heart rate change is a physiological indicator, which is extracted from cardiac patterns over short time periods (a few seconds). Heart rate change has been used extensively to explore orienting and defensive responses in aversive, threatening, and challenging situations (Bradley, 2009; Campbell et al., 1997; Gladwin et al., 2016; Graham & Clifton, 1966; Hermans et al., 2013; Kastner‐Dorn et al., 2018; Ribeiro & Castelo‐Branco, 2019). Interestingly, defensive responses have been associated with singular cardiac patterns that vary with the degree of proximity to the threat (Fanselow, 1994; Lang et al., 1997). Indeed, a freezing response was observed by cardiac deceleration (ECR1) when the threat was relatively distant or absent and cued, whereas a flight‐or‐fight response, also considered a stress response (Cannon, 1915), was visible by cardiac acceleration (ECR2) when the threat was closer. Functionally, freezing enhances the perception of a threat cue (Bradley, 2009) and prepares for action (Beggiato et al., 2019; Rösler & Gamer, 2019) through dominance of the parasympathetic autonomic component (Roelofs, 2017). In contrast, flight‐or‐fight promotes action through sympathetic activation and parasympathetic withdrawal (Schauer & Elbert, 2015). The substantial literature on defensive responses has demonstrated that these responses are well characterized by the cardiac components ECR1 and ECR2. Therefore, spotting these cardiac components during driving would be particularly advantageous for identifying the stress response (observed by ECR2) and anticipatory response to road hazards (revealed by ECR1, then ECR2) over short time periods.

### 1.3 Anticipation in driving and memorized situations

**FIGURE 1:**
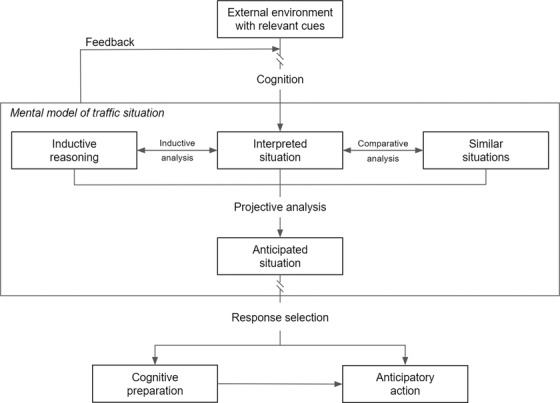
Adaptation and simplification of the model of anticipation in driving (Stahl et al., 2014)

Based on the model of anticipation in driving, Stahl et al. (2014) suggested that anticipatory responses (cognitive preparation and actions) were strongly guided by past experience of similar situations stored in episodic memory (see Figure 1). Indeed, the authors pointed out that increased anticipation could be explained by a “heightened ability to identify indicative cues, and interpret these cues relative to similar, memorized situations.” Therefore, if memorized and familiar situations improve drivers’ anticipation, this could also modify the underlying physiological responses, and thus strengthen the cardiac component ECR1 (i.e., increase the magnitude of cardiac deceleration). Such a physiological indicator would be of major interest to inform about the extent of driver's anticipatory response and, subsequently, to design alerts to improve anticipation of hazards.

### 1.4 Objectives

A literature search revealed a wide range of divergent definitions of stress (Lanatà et al., 2014). This paper is based on Selye's (1936) original definition, where *stress* refers to an adaptation phenomenon of the organism allowing living beings to react to hazardous events or situations. The first goal of the current study was to determine whether measuring heart rate change over short time windows (a few seconds) would detect driver stress caused by simulated hazardous road events. The second goal of this study was to explore whether heart rate change might provide a physiological signature of hazard anticipation in driving and depicted by the cardiac components ECR1 and ECR2. As the greater the anticipation, the greater the predictability, the signature of hazard anticipation was explored by manipulating hazard predictability. Thus, three levels of hazard predictability—unpredictable (U), unfamiliar and predictable (P), and familiar and predictable (PF)—were used to produce a non‐anticipatory response, an anticipatory response, and an increased anticipatory response, respectively.

- We expected that predictable events (P and PF) would shape a biphasic cardiac pattern before conflict with the event, thus reflecting an anticipatory response. This signature would consist of the cardiac component ECR1 (freezing response) indicating a cognitive preparation, and the cardiac component ECR2 (flight/fight response) indicating an anticipatory motor action,
- We hypothesized that a predictable and familiar event (PF), and thus memorized, would shape a greater cardiac component ECR1, thus reflecting an increased anticipation.
- We assumed that drivers perceiving events as highly stressful would reveal a lower magnitude of the cardiac component ECR1 than those perceiving them as less stressful, thus reflecting reduced anticipation.

## 2 METHODS

### 2.1 Participants

The experiment included 34 participants. However, only 27 participants (6 women, 21 men) aged between 21 and 51 years (M = 32.51; SD = 9.26) with at least 3 years of driving experience (M = 13.8; SD = 9.30) were included in the final analysis. Indeed, seven participants were excluded either because they did not complete the experiment due to motion sickness (five participants) or because the quality of the electrocardiogram recording was not satisfactory (two participants). All participants had normal or corrected‐to‐normal vision and declared no cognitive disorders and no heart disease. Participants were asked to not drink coffee or tea or consume sugar during the 2 h prior to the experiment in order to avoid disrupting their heart rate responses. All participants signed an informed consent form stating that electrocardiogram signals and driving information would be collected throughout the driving simulator experiment.

### 2.2 Study design and procedure

**FIGURE 2:**
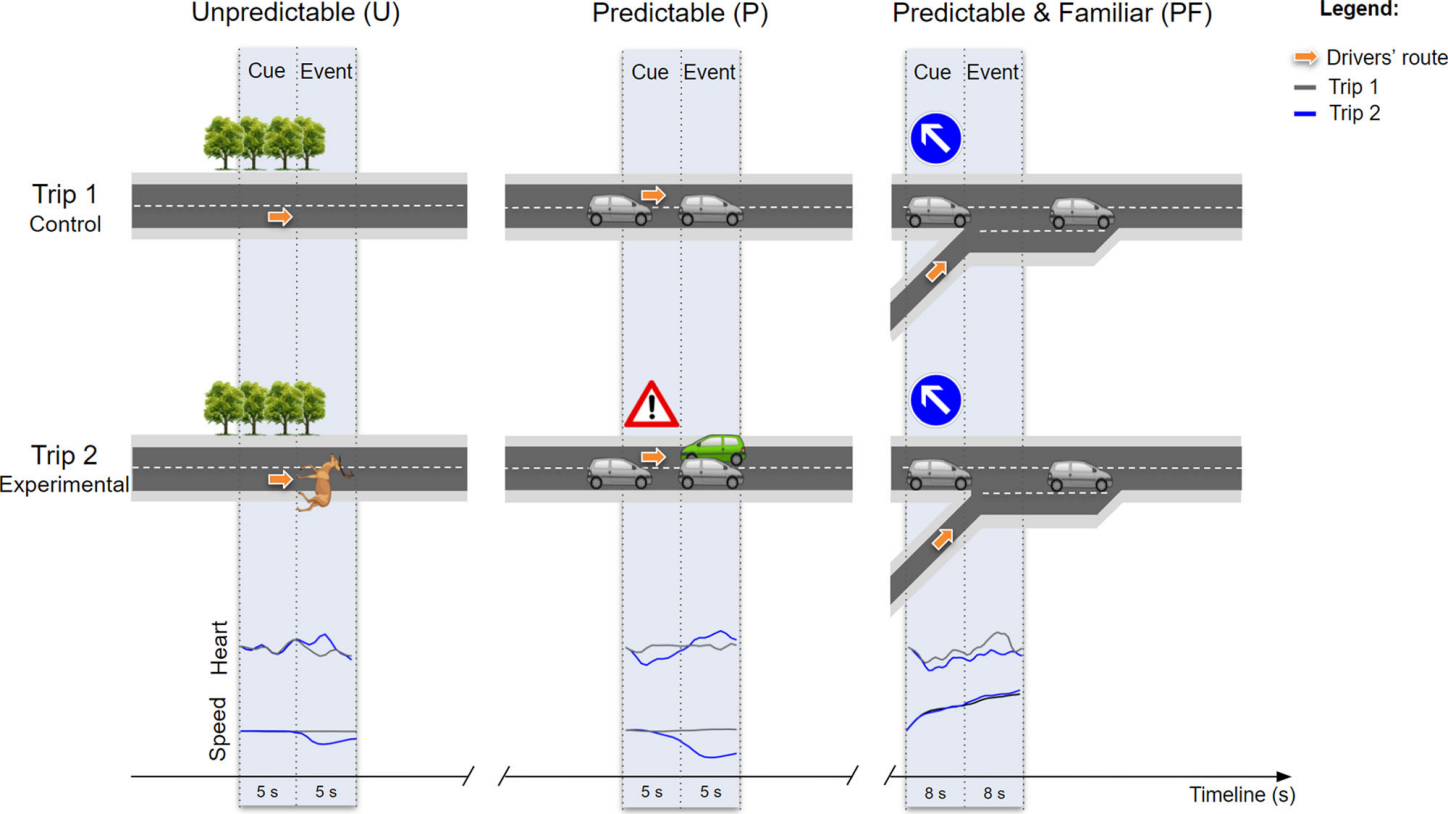
Overview of the drivers’ route in Trip 1 (control conditions) and Trip 2 (experimental conditions), as well as the mean changes in heart rate and speed for unpredictable (U), predictable (P), and predictable and familiar (PF) hazardous events, analyzed over two time windows (postcue and postevent)

The driving simulator study included three successive drives. For all drives, participants were instructed to comply with traffic laws and drive as they usually did. First, participants started with a training drive to become familiar with the driving simulator. Second, they drove on a simulated route for 10 min and the trip was recorded to provide control conditions (Trip 1; see Figure 2). Third, they drove for 10 min on the same simulated route as Trip 1 but this time under experimental conditions to permit the investigation of increased hazard predictability (Trip 2). Experimental conditions included three levels of predictability—an unfamiliar and unpredictable hazardous event (U), an unfamiliar and predictable hazardous event (P), and a familiar and predictable hazardous event (PF). We labeled “familiar” an event that was experienced in the past. Trip 1 was experienced prior to Trip 2 to prevent the drivers from forming expectations.

*Unpredictable condition*. The cue, regarded as a “foreshadowing element” of the upcoming hazard, was invisible to drivers (i.e., cue offset) in order to make the event surprising and prevent anticipation. The hazardous event, which took the form of a deer crossing the road after emerging from the forest, lasted 1.83 s (SD = 0.12) on average (i.e., from onset to offset).

*Predictable condition*. To make the event predictable, drivers were proactively alert of the upcoming hazard by a cue displayed prior to the hazardous event (i.e., cue onset). The cue, presented as an alert message on a simulated phone, lasted 4.41 s (SD = 0.42) on average, while the hazardous event, which took the form of an oncoming car on the wrong side of the road, lasted 3.01 s (SD = 0.40) on average.

*Predictable and familiar condition*. Likewise, a cue was displayed prior to the hazardous event to make the event predictable (i.e., cue onset). The cue, presented as a road sign on the dashboard informing the driver of the possibility of merging traffic, lasted 7.24 s (SD = 2.80) on average. The hazardous event, which took the form of a vehicle joining a busy highway, started at the onset of the highway access lane and lasted 3.73 s (SD = 1.49) on average. The cue and hazardous event were also presented in the control condition (Trip 1) to make the predictable event “familiar” in the experimental condition (Trip 2), thus allowing the study of increased hazard predictability.

Heart rate and speed data were continuously collected throughout the experiment but were analyzed specifically in the U, P, and PF conditions over two time windows—postcue and postevent. These two time windows were defined to permit the broad‐based study of the effect of hazard predictability (postcue) and of hazard exposure (postevent). At the end of the experiment, drivers were asked to report perceived stress for each hazardous event (Trip 2) compared to the corresponding safe condition (Trip 1). Perceived stress was assessed using 5‐point Likert stress scales, ranging from 1 “not stressful at all” to 5 “extremely stressful.” As no safe condition was available in Trip 1 for the predictable and familiar hazardous event (PF), perceived stress was not assessed for this condition.

### 2.3 Apparatus

**FIGURE 3:**
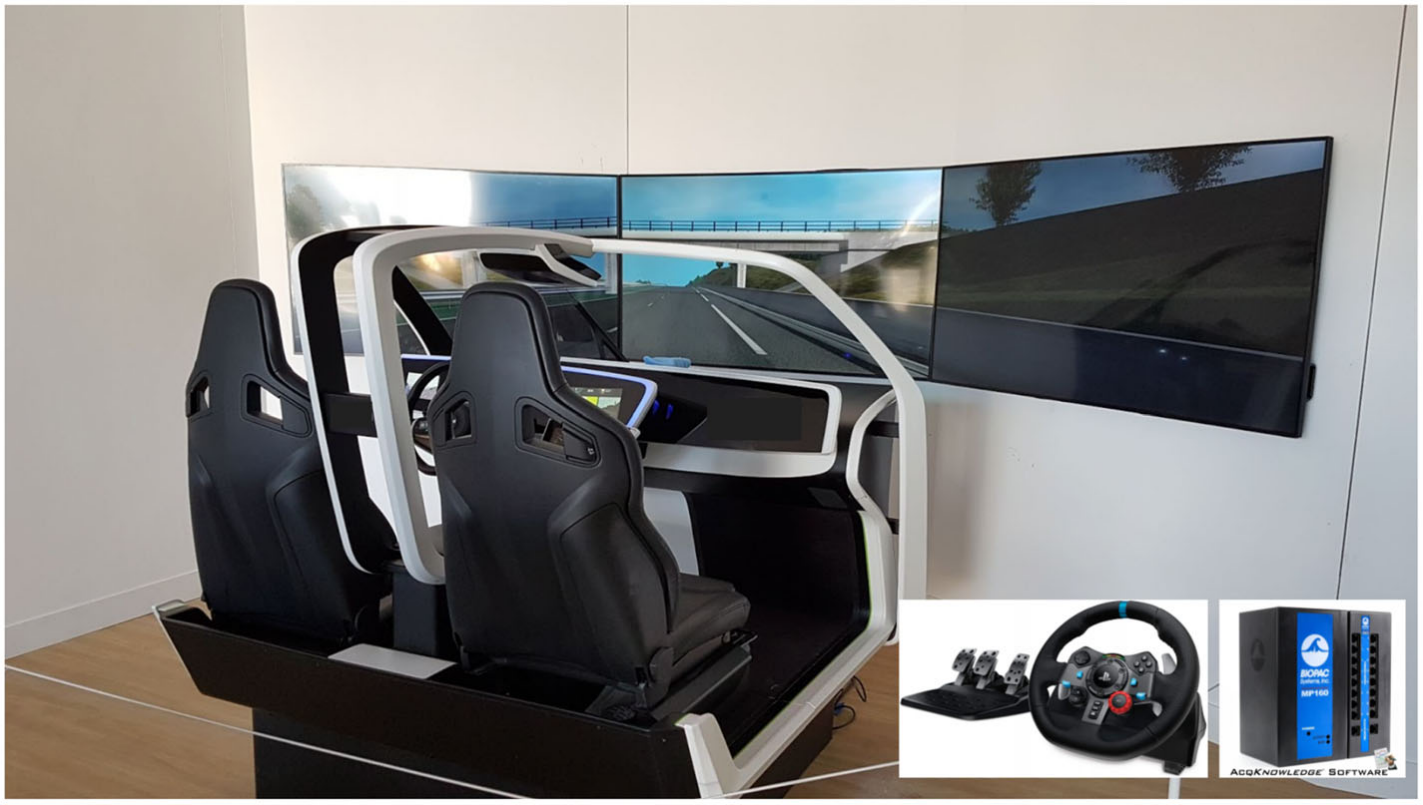
Overview of the equipment: homemade driving simulator, Biopac MP160 data acquisition system, Logitech G29 steering wheel and pedals

The experiment was performed in a homemade, fixed‐base driving simulator (see Figure 3) with a fully equipped interior: automatic gearbox, steering wheel and pedals (Logitech G29) as well as three screens (horizontal field of view: 130°) including a rear‐view mirror and two side mirrors. Unity 3D software was used to design the simulated driving environment. A physiological data acquisition system (BIOPAC MP160) was set up to collect drivers’ cardiac responses with a sampling rate of 500 Hz. To do this, two electrodes were placed on the manubrium of the sternum and the left lower rib cage, while the reference electrode was placed on the driver's right side at the top of the hip (Pépin et al., 2017). Driving speed and simulation‐related data (e.g., time markers for cues and hazardous events) were collected at a sampling rate of 10 Hz. Finally, Rtmaps software was used to time‐stamp, record, and synchronize the data from the different sensors.

### 2.4 Measurement, data processing, and statistical analysis

#### 2.4.1 Heart rate

Cardiac data were preprocessed using the same methodology as a previous research (Pépin et al., 2017). Each step is detailed below: (1) Electrocardiogram signals were band‐pass filtered, between 2 and 40 Hz, to filter extraneous noise, (2) R–R peaks were detected using an automatic detection procedure (AcqKnowledge 5.0 software), then extracted after visually checking the electrocardiogram signals for artifacts and correcting whenever R‐wave triggers were misplaced or omitted, (3) R–R intervals were converted into heart rate (beats per minute) as follows:

(1) $$\mathrm{RR}I_{n}=r_{n}-r_{n-1},$$

(2) $$HR_{n}=60/\mathrm{RR}I_{n},$$

where RRI*_n_* is the interval between two R‐wave peaks at a given time, in milliseconds, *r_n_* is the time corresponding to the *n*th R peaks, in milliseconds, and HR*_n_* is the heart rate corresponding to the *n*th R peaks, in beat per minute. (4) Using a cubic spline interpolation, heart rate data were sampled every 0.5 s for a period of −0.5 before and up to a maximum of 7.5 s after cue onset and after event onset. The heart rate value in the last half‐second prior to cue onset and event onset was used as a baseline for the postcue and postevent heart rate values, respectively. (5) Finally, heart rate change was calculated by subtracting the baseline from each postbaseline heart rate value as follows:

(3) $$\mathrm{HRC}\left(t\right)=\mathrm{HR}\left(t\right)-\mathrm{HR}\left(t_{0}\right),$$

where HRC(*t*) is the heart rate change at any given time in the time window, in beat per minute, HR(*t*) is the heart rate at any given time in the time window, in beat per minute, and HR(*t*_0_) is the heart rate at the beginning of the time window, in beat per minute.

Heart rate change was analyzed by running separate repeated‐measures ANOVAs; one for each time window (postcue and postevent) within each hazard predictability condition (U, P, and PF). Each ANOVA included the condition‐type (experimental vs. control) and time point (10 levels for U and P and 16 levels for PF) as within‐subject factors. Greenhouse–Geisser corrections were applied when there was a sphericity violation for time. Post hoc comparisons were performed by applying Bonferroni corrections. Effect sizes were estimated using partial eta‐squared (*η* ^2^_p_) for ANOVAs and Cohen's *d* for pairwise comparisons. For all the analyses, the significance level α was set at *p* < .05.

Furthermore, given our assumptions about the cardiac pattern during hazard anticipation, we used polynomial contrasts to determine whether the change in heart rate followed the expected mathematical pattern over time. For this reason, polynomial contrasts were performed only for conditions that included a predictable hazardous event and only for the postcue time window. According to Lawrence and Barry (2009), a brief phasic cardiac response (i.e., reflected by an initial cardiac deceleration then by a slightly later cardiac acceleration) is revealed by a quadratic trend over a short time window.

#### 2.4.2 Speed

Speed data were converted to speed change using the same methodology as for heart rate change. Statistical analyses of speed change were then performed using separate repeated‐measures ANOVAs, in the same way as for heart rate change.

#### 2.4.3 Perceived stress

For statistical analyses of perceived stress‐modulated driver responses, we first formed two groups using the median split; drivers who rated the hazardous event (relative to the corresponding safe condition) above 2.5 out of 5 were assigned to the high‐perceived stress group, while the others were placed in the low‐perceived stress group. The high‐ and low‐perceived stress groups consisted of 11 and 16 drivers, respectively, for U, and 13 and 14 drivers for P.

Then, for each perceived stress group, we calculated areas under the curve (AUC) for the control and experimental conditions in order to represent changes in drivers’ response (heart rate or speed) averaged over a time window (postcue or postevent). We then used paired *t*‐tests to compare AUC between experimental and control conditions within each perceived stress group. Effect sizes were estimated using Cohen's *d*.

## 3 RESULTS

### 3.1 Unpredictable condition

**FIGURE 4:**
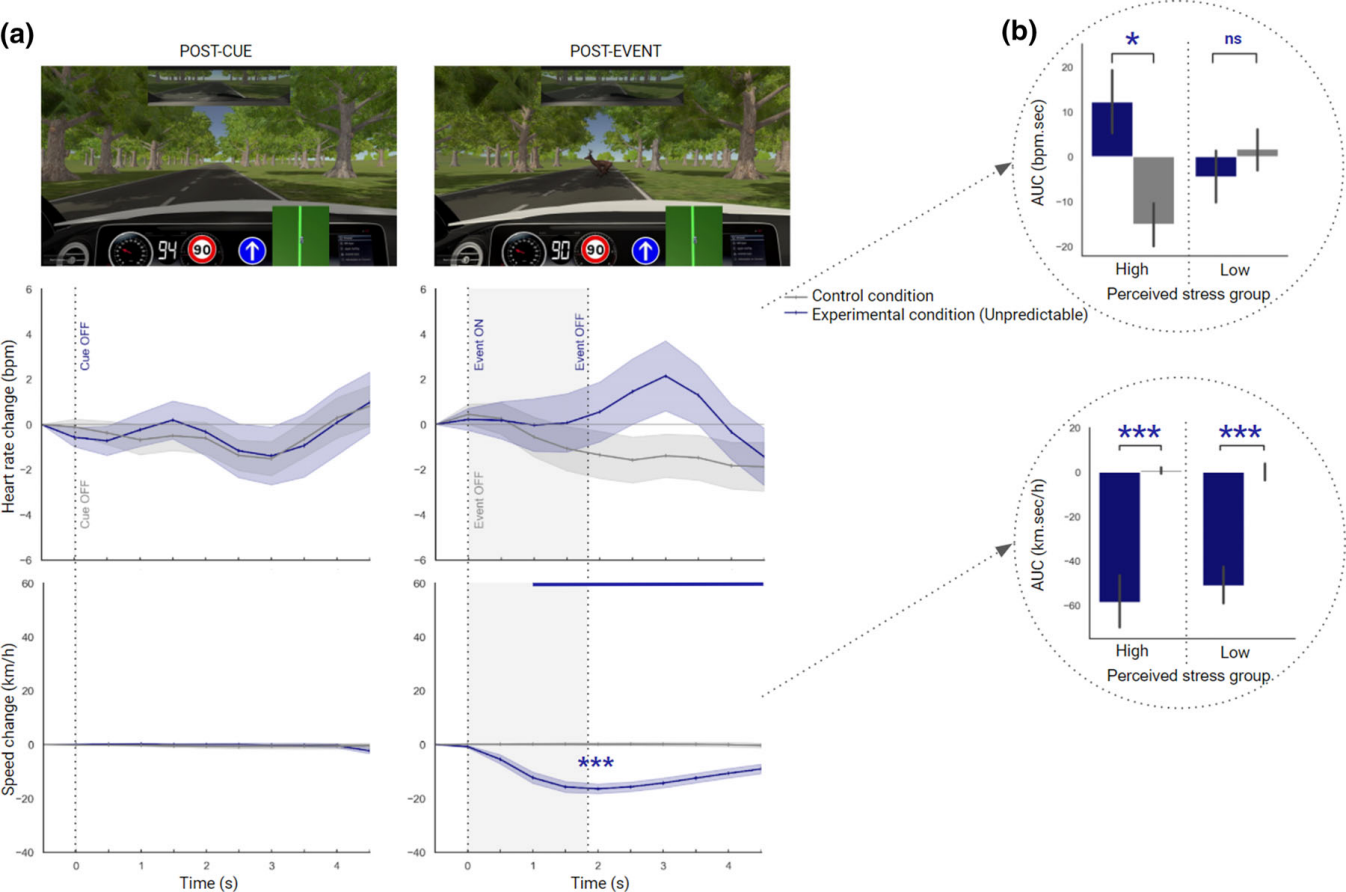
(a) Changes in heart rate and speed relative to the last half‐second prior to cue‐onset and event‐onset when drivers experienced the control condition (gray) and the unpredictable (U) experimental condition (blue). Cues and events were either ON (visible) or OFF (invisible). Shaded areas denote standard errors of the mean. An overall significant difference between conditions is represented by blue asterisks as follows: *p < .05; **p < .01; ***p < .001. Time points with significant differences between the experimental and control conditions are displayed at the top of each figure as a blue horizontal line. The pictures depict cue OFF (left‐side) and event ON (right‐side). (b) Changes in heart rate and speed, averaged over the post‐event time window, for the control and experimental conditions, in the high‐ and low‐perceived stress groups

We first ran repeated‐measures ANOVAs to compare heart rate in the experimental condition (including an unpredictable hazardous event) with the control condition (safe) in the postcue time window, and in the postevent time window (Figure 4(a)). Next, we repeated the process with speed. Heart rate and speed were finally investigated in each perceived stress group (Figure 4(b), Table 1).

**TABLE 1:** Heart rate and speed for the high‐ and low‐perceived stress groups within the unpredictable (U) and predictable (P) conditions in the postcue and postevent time windows

				Postcue			Postevent		
Hazard predictability condition	Response	Perceived stress group	*N*	Experimental AUC	Control AUC	*p*‐value (*d*)	Experimental AUC	Control AUC	*p*‐value (*d*)
Unpredictable (U)	Heart rate	High	11	−9.55 (30.2)	−5.81 (9.94)	.71 (−0.11)	12.2 (24.5)	−15.0 (16.2)	<.05* (0.73)
		Low	16	2.76 (14.7)	1.14 (14.0)	.75 (0.08)	−4.49 (25.6)	1.70 (18.5)	0.40 (−0.21)
	Speed	High	11	−6.42 (8.76)	2.43 (7.52)	.072 (−0.60)	−58.7 (39.2)	0.82 (4.78)	< .001^***^ (−1.47)
		Low	16	3.27 (10.7)	−5.92 (17.6)	.11 (0.42)	−51.2 (34.8)	0.38 (16.1)	< .001^***^ (−1.18)
Predictable (P)	Heart rate	High	13	−9.65 (21.7)	1.43 (13.4)	.15 (−0.41)	18.6 (5.2)	−0.28 (23.7)	<.05* (0.71)
		Low	14	−8.92 (10.7)	0.82 (20.0)	.16 (−0.39)	2.48 (23.9)	−5.86 (15.9)	0.31 (0.27)
	Speed	High	13	−26.1 (19.0)	−8.47 (−22.8)	.06 (−0.56)	−129.2 (100.3)	−5.10 (20.7)	< .01** (−1.09)
		Low	14	−11.5 (42.6)	−0.17 (28.3)	.44 (−0.20)	−104.4 (85.9)	17.12 (48.19)	< .001^***^ (−1.43)

*Note*. *N* = number of drivers; AUC = mean area under the curve and standard deviation (in parentheses); *p*‐value (*d*) = level of significance and effect size (in parentheses) for each difference between experimental and control conditions.

#### 3.1.1 Heart rate

*Postcue*. The ANOVA showed no significant main effect of condition‐type (*F*_(1, 26)_ = 0.011, *p* = .917, *η* ^2^_p_ = 4.24e‐4), no main effect of time (*F*_(2.50, 65.21)_ = 2.51, *p* = .076, *η* ^2^_p_ = .088), and no condition‐type × time interaction (*F*_(2.52, 65.73)_ = 0.106, *p* = .936, *η* ^2^_p_ = .004), suggesting that heart rate remained unchanged overall in both the experimental and control conditions.

*Postevent*. The ANOVA revealed no significant main effect of condition‐type (*F*_(1, 26)_ = 1.08, *p* = .307, *η* ^2^_p_ = .040), no main effect of time (*F*_(3.69, 92.45)_ = 2.78, *p* < .05*, *η* ^2^_p_ = .100), and no condition‐type × time interaction (*F*_(2.73, 71.08)_ = 1.74, *p* = .171, *η* ^2^_p_ = .063), indicating that heart rate change in the experimental condition was not statistically different from in the control condition.

#### 3.1.2 Speed

*Postcue*. The ANOVA showed no significant main effect of condition‐type (*F*_(1, 26)_ = 0.083, *p* = .776, *η* ^2^_p_ = .003), no main effect of time (*F*_(1.36, 35.48)_ = 1.91, *p* = .173, *η* ^2^_p_ = .068), and no condition‐type × time interaction (*F*_(1.76, 45.93)_ = 1.98, *p* = .154, *η* ^2^_p_ = .071), reflecting a constant speed in both experimental and control conditions.

*Postevent*. The ANOVA revealed a significant main effect of condition‐type (*F*_(1, 26)_ = 44.71, *p* < .001*** , *η* ^2^_p_ = .632), a main effect of time (*F*_(2.71, 70.46) _= 30.92, *p* < .001*** , *η* ^2^_p_ = .543), as well as a significant condition‐type × time interaction (*F*_(2.33, 60.72)_ = 28.00, *p* < .001*** , *η* ^2^_p_ = .519). This result suggests that speed was lower overall in the experimental condition than in the control condition (*t* = −6.687, *p* < .001***, *d* = −1.287), and also that the experimental condition was associated with a marked decrease from 1 s (Figure 4(a), see also Table S1 for statistics of post hoc tests over time).

#### 3.1.3 Perceived stress

*Global*. For all drivers, the mean perceived stress score for the unpredictable hazardous event was 2.30 (SD = 1.03) out of 5.

*Per group*. The mean perceived stress score per group was 3.36 (SD = 0.50) and 1.56 (SD = 0.51) for the high‐ and low‐perceived stress groups, respectively. Our investigation of drivers’ responses in each group revealed significant effects only in the post‐event time window (Table 1). Paired *t*‐tests indicated, first, that heart rate was significantly greater in the experimental condition than in control condition only for the high‐perceived stress group (*p* < .05*, *d* = 0.733), and, second, that speed was significantly lower in the experimental condition than in control condition for both the high‐ and low‐perceived stress groups (high, *p* < .001***, *d* = −1.471, low, *p* < .001***, *d* = −1.185) (Figure 4(b), Table 1).

### 3.2 Predictable condition

**FIGURE 5:**
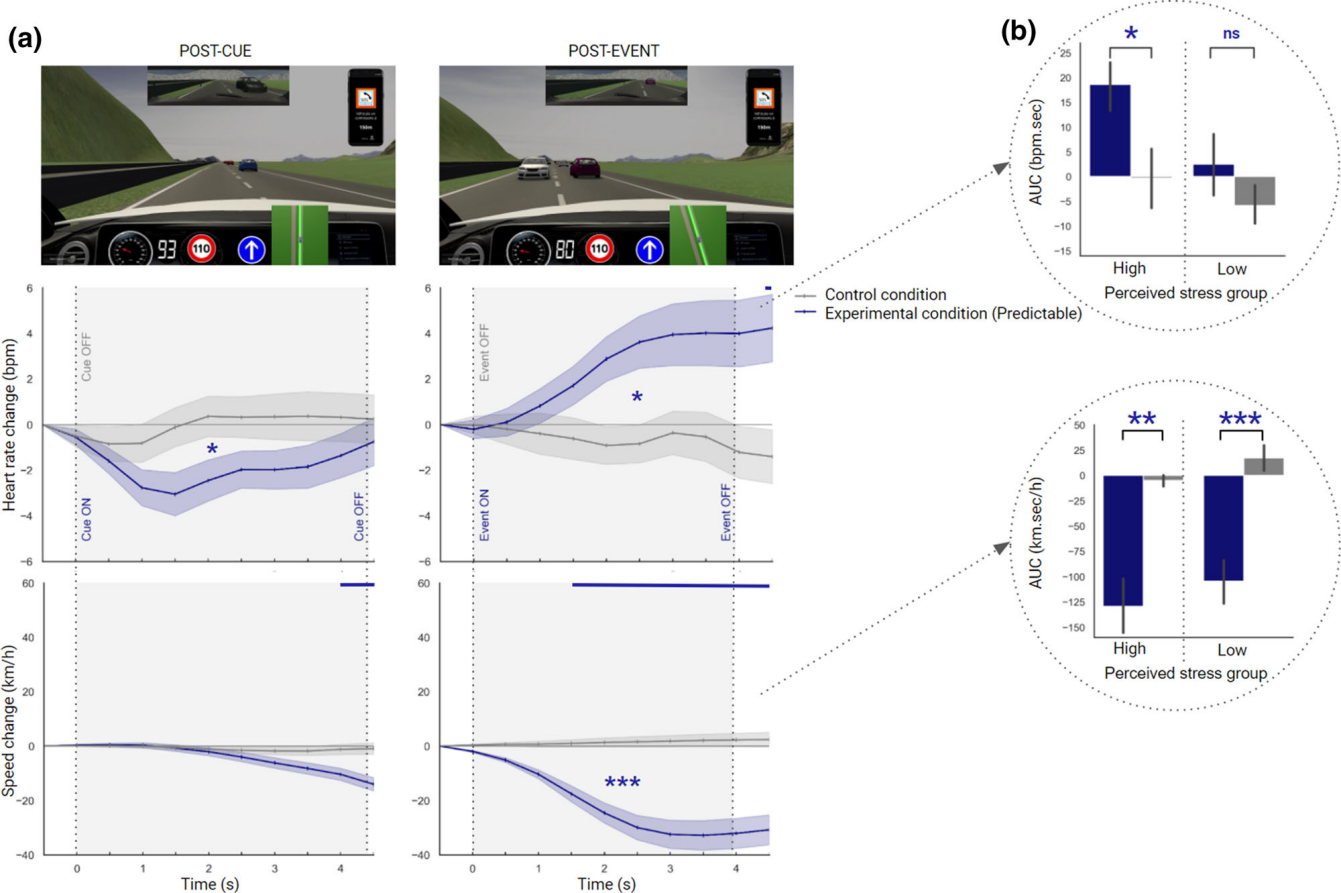
(a) Changes in heart rate and speed relative to the last half‐second prior to cue‐onset and event‐onset when drivers experienced the control condition (gray) and the predictable (P) experimental condition (blue). Cues and events were either ON (visible) or OFF (invisible). Shaded areas denote standard errors of the mean. An overall significant difference between conditions is represented by blue asterisks as follows: *p < .05; **p < .01; ***p < .001. Time points with significant differences between the experimental and control conditions are displayed at the top of each figure as a blue horizontal line. The pictures depict cue ON (left‐side) and event ON (right‐side). (b) Changes in heart rate and speed, averaged over the post‐event time window, for the control and experimental conditions, in the high‐ and low‐perceived stress groups

We first used repeated‐measures ANOVAs to compare heart rate in the experimental condition (including a predictable hazardous event) with the control condition (safe) in the postcue time window, and in the postevent time window (Figure 5(a)). Then, we repeated the process with speed. Heart rate and speed were finally investigated in each perceived stress group (Figure 5(b), Table 1).

#### 3.2.1 Heart rate

*Postcue*. The ANOVA yielded a significant main effect of condition‐type (*F*_(1, 26)_ = 4.26, *p* = .049*, *η* ^2^_p_ = .141). In contrast, no significant main effect of time (*F*_(2.98, 77.69)_ = 1.79, *p* = .156, *η* ^2^_p_ = .064) and no condition‐type × time interaction (*F*_(2.57, 26)_ = 1.62, *p* = .197, *η* ^2^_p_ = .059) were found, thus suggesting that heart rate change was overall lower in the experimental condition than in the control condition (*t* = −2.066, *p* < .05*, *d* = −0.398) (Figure 5(a)). In addition, polynomial contrasts indicated that the cardiac pattern from the experimental condition fitted a quadratic trend (*p* < .001***) (Table 2).

**TABLE 2:** Outcomes of mathematical cardiac pattern analysis corresponding to the predictable (P) and, predictable and familiar (PF) conditions in the postcue time window

			Postcue	
Hazard predictability condition		Trend of cardiac pattern	*t*	*p*‐value
Predictable (P)	Experimental	Linear	−0.82	.411
		Quadratic	4.79	<.001^***^
		Cubic	−1.26	.207
Predictable and Familiar (PF)	Experimental	Linear	−1.93	.054
		Quadratic	5.50	<.001^***^
		Cubic	−1.31	.190
	Control	Linear	0.14	.88
		Quadratic	3.92	<.001^***^
		Cubic	−1.80	.071

*Note*. Polynomial contrasts were performed only for conditions including a hazard cue.

*Postevent*. The ANOVA highlighted a significant main effect of condition‐type (*F*_(1, 26)_ = 7.07, *p* < .05*, *η* ^2^_p_ = .214) and a condition‐type × time interaction (*F*_(2.69, 70.11)_ = 6.36, *p* = .001*** , *η* ^2^_p_ = .197). In contrast, no main effect of time (*F*_(1.91, 49.77)_ = 2.54, *p* = .091, *η* ^2^_p_ = .089) was found, indicating that heart rate change was globally higher in the experimental condition than in the control condition (*t* = 2.661, *p* < .05*, *d* = 0.512), and also that the experimental condition was associated with a gradual cardiac acceleration, as evidenced by a significant threshold reached at 4.5 s after the onset of the predictable hazardous event (Figure 5(a), see also Table S1).

#### 3.2.2 Speed

*Postcue*. The ANOVA showed a significant main effect of time (*F*_(1.76, 45.79)_ = 21.53, *p* < .001***, *η* ^2^_p_ = .453) and condition‐type × time interaction (*F*_(1.40, 36.63)_ = 13.69, *p* < .001***, *η* ^2^_p_ = .345). There was, however, no significant main effect of condition‐type (*F*_(1, 26)_ = 3.75, *p* = .064, *η* ^2^_p_ = .126), suggesting that changes in speed differed between conditions over the evolving time course of the experiment. Indeed, a substantially greater speed deceleration was revealed in the experimental condition as of 4 s after cue onset (Figure 5(a); see also Table S1).

*Postevent*. The ANOVA showed a significant main effect of condition‐type (*F*_(1, 26)_ = 42.49, *p* < .001***, *η* ^2^_p_ = .620), a main effect of time (*F*_(1.28, 33.36)_ = 19.64, *p* < .001***, *η* ^2^_p_ = .430), and a time × condition‐type interaction (*F*_(1.33, 34.78) _= 28.39, *p* < .001***, *η* ^2^_p_ = .522). This result revealed that speed was globally lower in the experimental condition than in the control condition (*t* = −6.518, *p* < .001***, *d* = −1.254), and also that the experimental condition was associated with a marked decrease from 1.5 s after event onset (Figure 5(a); see also Table S1).

#### 3.2.3 Perceived stress

*Global*. The mean perceived stress score among all drivers for the predictable hazardous event was 2.67 (SD = 1.62) out of 5.

*Per group*. The mean perceived stress score per group was 4.19 (0.80) and 1.25 (SD = 0.42) for high‐ and low‐perceived stress groups, respectively. Our examination of drivers’ responses in each group revealed only a significant effect in the postevent time window (Table 1). Paired *t*‐tests revealed, first, that heart rate was significantly greater in the experimental than in the control condition for the high‐perceived stress group (*p* < .05*, *d* = 0.715), and, second, that speed was significantly lower in the experimental condition than in control condition for both the high‐ and low‐perceived stress groups (high, *p* < .01**, *d* = −1.094, low, *p* < .001***, *d* = −1.433) (Figure 5(b), Table 1).

### 3.3 Predictable and familiar condition

**FIGURE 6:**
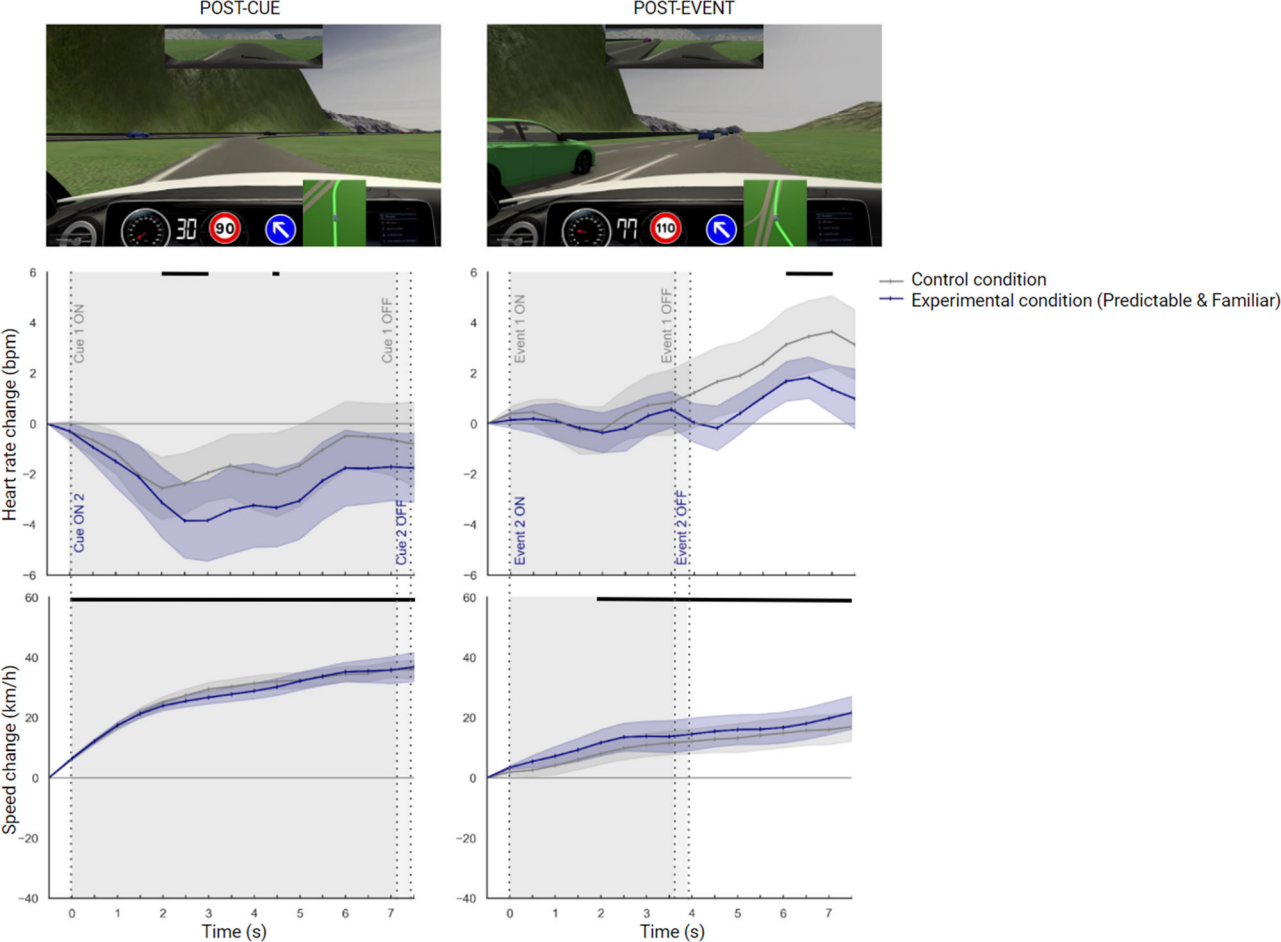
Changes in heart rate and speed relative to the last half‐second prior to cue‐onset and event‐onset when drivers experienced the control condition (gray) and the predictable and familiar (PF) experimental condition (blue). Cues and events were systematically ON (visible). Shaded areas denote standard errors of the mean. Time points with significant differences relative to baseline (*t* = −0.5 s) are displayed at the top of each figure as a black horizontal line. The pictures depict cue ON (left‐side) and event ON (right‐side)

We first used repeated‐measures ANOVAs to compare heart rate in the experimental condition (including a predictable and familiar hazardous event) with the control condition (including a predictable hazardous event) in the postcue time window, and in the postevent time window (Figure 6). Second, we repeated the process with speed.

#### 3.3.1 Heart rate

*Postcue and Postevent*. The ANOVAs indicated no significant main effect of condition‐type (Postcue, *F*_(1, 26)_ = 0.39, *p* = .536, *η* ^2^_p_ = .015, Postevent, *F*_(1, 26)_ = 0.50, *p* = .482, *η* ^2^_p_ = .019) and no condition‐type × time interaction (Postcue, *F*_(2.93, 76.18)_ = 0.40, *p* = .743, *η* ^2^_p_ = .015, Postevent, *F*_(2.72, 70.94) _= 0.60, *p* = .597, *η* ^2^_p_ = .023). There was, however, a significant main effect of time (Postcue, *F*_(2.24, 58.32)_ = 3.33, *p* < .05*, *η* ^2^_p_ = .11, Postevent, *F*_(3.81, 99.2)_ = 5.55, *p* < .001***, *η* ^2^_p_ = .17), suggesting that the experimental and control conditions evoked similar changes in heart rate over time in both time windows. In addition, for both conditions, a strong cardiac deceleration was revealed between 2 s and 3 s, as well as at 4.5 s in the postcue time window, and a strong cardiac acceleration was found between 6 s and 7 s in the postevent time window (Figure 6; see also Table S1). Additionally, polynomial contrasts performed in the postcue time window indicated that cardiac patterns in the experimental and control conditions both followed a quadratic trend (*p* < .001***) (see Table 2).

#### 3.3.2 Speed

*Postcue and Post‐event*. The ANOVAs indicated a significant main effect of time (Cue, *F*_(2.69, 69.96)_ = 76.64, *p* < .001***, *η* ^2^_p_ = .74, Event, *F*_(2.31, 60.13)_ = 10.49, *p* < .001***, *η* ^2^_p_ = .28), but no main effect of condition‐type (Cue, *F*_(1, 26)_ = 0.069, *p* = .795, *η* ^2^_p_ = .003, Event, *F*_(1, 26)_ = 0.27, *p* = .604, *η* ^2^_p_ = .011) and no interaction between condition‐type and time (Cue, *F*_(2.01, 52.28)_ = 0.30, *p* = .741, *η* ^2^_p_ = .012, Event, *F*_(2.31, 60.26)_ = 0.21, *p* = .918, *η* ^2^_p_ = .004), suggesting that the experimental and control conditions elicited similar changes in speed over time in both time windows. Also, for both conditions, strong speed accelerations were found between 0 s and 7.5 s in the postcue time window, and between 2 s and 7.5 s in the postevent time window (Figure 6; see Table S1).

## 4 DISCUSSION

### 4.1 Driver stress detection

The first purpose of the current study was to determine whether using heart rate change over short time windows would make it possible to detect driver stress. To this end, driver stress was manipulated using simulated hazardous road events.

The participants reported that the perceived stress related to the hazardous events was moderate overall. Indeed, the average scores for the events U and P were 2.30 (SD = 1.03) and M = 2.67 (SD = 1.62) out of 5, respectively. These data therefore suggest that stress was successfully induced by the hazardous events. Only the event PF was not subjectively assessed as there was no corresponding safe condition for comparison. Given the absence of such a condition, we simulated the event PF to be as stressful as possible by implementing a busy road traffic situation with a short merging lane. As a result, it seemed reasonable to us to consider that stress was perceived by the drivers and to gauge the effect of this stress solely on the basis of the cardiac response.

When we examined the cardiac responses after the onset of the hazardous events U, P, and PF, an increase in heart rate was systematically observed either for all drivers (conditions P and PE) or for a part of them (condition U). Cardiac acceleration is consistent with the idea of flight‐or‐fight response, when the hazard is proximal (Lang et al., 1997). Moreover, the drastic speed deceleration associated with cardiac acceleration after the onset of avoidable hazardous events (U and P) supports the evidence of flight response, and thus stress expression.

By way of comparison, previous studies also found an increase in heart rate after using simulated hazardous road events (Johnson et al., 2011; Schmidt‐Daffy, 2013). In contrast, Gemonet et al. (2021) did not observe such an effect, and more surprisingly, they reported a change in cardiac response under real driving conditions using the same road event and testing the same individuals as in the simulator. Based on these studies, we suspect that some road events may not be sufficiently threatening to induce driver stress in a driving simulator. Also, it is possible that the lack of safety concerns in an artificial driving environment can mitigate the stressful and threatening effects of some road events.

In addition, it should be noted in our study that cardiac acceleration for the unpredictable hazardous event (U) was significant only in the high‐perceived stress group, but was not globally so. This result thus contrasts with a previous study in which global cardiac acceleration was observed after the onset of unpredictable road events over a short time period (Johnson et al., 2011). Interestingly, global cardiac acceleration has also been found using the same type of hazardous simulated event as in our study, that is, a deer appearing on the road (Schmidt‐Daffy, 2013). In addition, the fact that cardiac acceleration after the onset of the predictable hazardous event (P) was also significant for the high but not for the low‐perceived stress group suggests that individual differences in stress perception and experience can definitively influence cardiac response.

In the light of our findings, we can confirm that it is possible to detect driver stress by using heart rate change over a short time period. Nevertheless, our results, along with those of previous studies, suggest that future studies should closely consider both the threat potential of simulated hazardous events and the subjective perception of stress in order to facilitate stress induction as well as its measurement.

### 4.2 Hazard anticipation detection and investigation

The second aim of the current study was to explore the physiological signature of hazard anticipation in driving using heart rate change. To this end, hazard anticipation was manipulated by means of three levels of hazard predictability (U, P, and PF). Three broad hypotheses were formulated.

#### 4.2.1 Hypothesis (i): Effects of predictability

Based on the theory that defensive responses are determined by proximity to the threat (Fanselow, 1994; Lang et al., 1997), we expected that predictable events (P and PF) would shape a biphasic cardiac pattern before conflict with the event. This pattern would consist of first the cardiac component ECR1 (freezing response) reflecting a cognitive preparation (attentional enhancement and planification of motor action), and second the ECR2 component (flight/fight response) representing an anticipatory motor action.

When the hazardous events were cued to drivers (P and PF), we observed similar and singular cardiac patterns after cue onset. Indeed, biphasic cardiac responses were observed including, first, an initial cardiac deceleration (ECR1) followed slightly later by cardiac acceleration (ECR2). Such biphasic patterns were highlighted by significant quadratic trends, thus supporting the presence during hazard anticipation of the cardiac components ECR1 and ECR2. Cardiac deceleration in a threatening automotive context has been previously interpreted as reflecting a need to increase visual information intake in order to cope with a threatening situation (Barnard & Chapman, 2016) and has been more generally linked to the phenomenon of preparation for action (Beggiato et al., 2018, 2019).

Specifically, after cue onset in the predictable condition (P), we observed, first, a biphasic cardiac response, marked by a peak at 1.5 s, and, second, a speed decrease also initiated at 1.5 s. As a result, an interesting visual correspondence can be noted between cardiac and speed responses. This correspondence is consistent with the theory of preparation/initiation of action (Gladwin et al., 2016; Lang & Davis, 2006; Rösler & Gamer, 2019). According to this theory, the decelerating cardiac component associated with the any speed change up to 1.5 s, would reflect an active state of “attentive immobility” (freezing response), while the accelerating cardiac component associated with a slightly later speed deceleration after 1.5 s, would suggest the initiation of action (flight response). These initial observations should therefore encourage the use of cardiac pattern analysis to determine whether drivers engage in cognitive preparation when they detect a hazard, and also to predict a subsequent motor action seconds before it is performed (e.g., flight behavior).

Furthermore, the conclusion that hazard predictability resulted in a biphasic cardiac signature, reflecting anticipation, is reinforced by the fact that hazard unpredictability did not. Indeed, when drivers received no cue of the upcoming hazard event (U), cardiac and speed responses remained unchanged.

#### 4.2.2 Hypothesis (ii): Effects of increased predictability

Based on the relationship between anticipation and memorized situations (Stahl et al., 2014), we hypothesized that a predictable and familiar event (PF) would shape a greater cardiac component ECR1, thus reflecting an increased anticipation of the hazard. However, our results indicated no change in heart rate between the predictable and familiar event (PF) and the predictable and unfamiliar event (first exposure). This lack of significance prevents us from validating the initial hypothesis that increased predictability would emphasize the magnitude of the cardiac component ECR1. Although a visual inspection of the cardiac patterns associated with the first and second exposures is consistent with this hypothesis, the fact that the results are nonsignificant may suggest that the event was not sufficiently familiar, experienced, and memorized for such a differentiation to be statistically observed. Therefore, it would be worthwhile in the future to repeat exposures to the same road hazard in order to clarify the effect of increased predictability and anticipation on cardiac changes.

#### 4.2.3 Hypothesis (iii): Effects of perceived stress

We assumed that drivers perceiving events as highly stressful would reveal a lower magnitude of the ECR1 component than those perceiving them as less stressful, thus reflecting reduced anticipation. Contrary to a previous study in which higher levels of subjective arousal was related with a shallower ECR1 cardiac component (Binder et al., 2005), we found after cue onset in the condition P no change in the magnitude of the ECR1 component according to the perceived stress group. However, cardiac responses were modulated by the perceived stress group after the exposure to the predictable hazardous event (P). Again, further investigation would be necessary to understand the relationship between perceived stress and changes in the magnitude of the cardiac components.

### 4.3 Limitations

Our experiment is subject to several limitations. First, the control conditions for U and P were systematically presented before the experimental conditions in which the hazardous events occurred. Thus, the order of the conditions was not counterbalanced to prevent a previously experienced hazardous event from inducing anticipatory stress in the control conditions. Therefore, our protocol did not control for learning and acclimatization effects related to simulated driving. Nevertheless, stress responses observed in the experimental conditions, cannot be methodological artifacts since we obtained opposite effects to those usually produced by learning and acclimatization. Indeed, previous research has shown that learning and acclimatization effects did not cause or increase driver stress, but rather reduced it (Chen, 2015; Heikoop et al., 2017). It should be noted that such effects have been observed using cardiac measurements as well.

A second limitation is that the conclusions about the effects of hazard anticipation and predictability on changes in heart rate and speed were drawn from different road events. Again, to avoid inducing anticipatory stress, we did not use an experimental design in which the predictability dimension was manipulated using the same hazardous event. Nevertheless, it would be wise to also use this approach in order to permit the generalization of the findings.

A third limitation concerns the limited potential of simulated hazard stimuli to elicit stress states. Indeed, drivers overall reported a moderate perceived stress for the event U (M = 2.30, SD = 1.03) and the event P (M = 2.67, SD = 1.62). Therefore, dynamically adjusting the triggering of simulated hazardous events based on drivers’ behaviors (e.g., speed, lane positioning) so that each participant experiences the event in the most stressful way possible could be an improvement that would increase perceived stress.

A fourth limitation is related to the phenomenon of respiratory sinus arrhythmia resulting in a cardiac acceleration during inhalation and cardiac deceleration during exhalation (Beauchaine et al., 2019). Previous works have found that removing respiratory influences from heart rate could improve the identification of vagal withdrawal and increase sympathetic activation (Choi & Gutierrez‐Osuna, 2011; Varon et al., 2018). Consequently, by adopting such an approach in the future, studies could fine‐tune the sensitivity of heart rate change to hazardous events.

A last limitation is that our conclusions are based on results obtained in a driving simulator. Although we mentioned in the introduction the ability of an artificial driving environment to highlight driver states such as stress, it cannot be excluded that the environment can influence the stress response. In this sense, heart rate was found to be higher during on‐road driving than during simulated driving (Johnson et al., 2011). Therefore, it would be interesting to explore cardiac patterns in real road studies to assess the extent of differences related to real and simulated environments, and especially to investigate the potential for application.

## 5 CONCLUSION

To summarize, this study demonstrates the value of using heart rate change to detect driver stress in real time. The use of heart rate change is also of major interest since it provides a physiological signature of hazard anticipation, as evidenced by a biphasic cardiac pattern. Further investigation is required to validate the lack of relationship between increased predictability/anticipation and increased cardiac component ECR1. We also observed that perceived stress modulated the cardiac response after exposure to hazardous events. However, further research is needed to confirm the lack of influence of perceived stress on the cardiac response to a hazard cue. These initial results may pave the way for further studies designed to explore changes in heart rate as a function of drivers' anticipation and preparation for hazardous road events. Such studies promise to make a valuable contribution to solutions that will proactively alert drivers to hazardous road events and, more generally, to the design of driver stress detection systems.

## CONFLICT OF INTEREST

The authors declare no conflict of interest.

### PEER REVIEW

The peer review history for this article is available at https://publons.com/publon/10.1002/brb3.2424

## Supporting information

Appendices

## Data Availability

The data that support the findings of this study are available from the corresponding author upon reasonable request.
