# Supplementary material for: Detecting driver stress and hazard anticipation using real‐time cardiac measurement: A simulator study
Source: Brain Behav. 2022 Jan 28;12(2):e2424. doi: 10.1002/brb3.2424 (PMC8865166; doi:10.1002/brb3.2424)
Supplement: Supplementary file 1 — Appendices [file BRB3-12-e2424-s001.docx]

Appendices

**Table S1**. Post-hoc tests per condition.

| Unpredictable condition (U) | | | | | | | | |
| --- | --- | --- | --- | --- | --- | --- | --- | --- |
| Time in seconds | | |  | Heart rate | |  | Speed | |
|  |  |  |  | Post-cue | Post-Event |  | Post-cue | Post-Event |
| experimental at t = 0 s | vs. | control at t = 0 s | *t* | - | - |  | - | -0.467 |
|  |  |  | *p* | - | - |  | - | 1.000 |
| experimental at t = 0.5 s | vs. | control at t = 0.5 s | *t* | - | - |  | - | -2.969 |
|  |  |  | *p* | - | - |  | - | 1.000 |
| experimental at t = 1 s | vs. | control at t = 1 s | *t* | - | - |  | - | -6.650 |
|  |  |  | *p* | - | - |  | - | **< .001***** |
| experimental at t = 1.5 s | vs. | control at t = 1.5 s | *t* | - | - |  | - | -8.495 |
|  |  |  | *p* | - | - |  | - | **< .001***** |
| experimental at t = 2 s | vs. | control at t = 2 s | *t* | - | - |  | - | -8.828 |
|  |  |  | *p* | - | - |  | - | **< .001***** |
| experimental at t = 2.5 s | vs. | control at t = 2.5 s | *t* | - | - |  | - | -8.483 |
|  |  |  | *p* | - | - |  | - | **< .001***** |
| experimental at t = 3 s | vs. | control at t = 3 s | *t* | - | - |  | - | -7.697 |
|  |  |  | *p* | - | - |  | - | **< .001***** |
| experimental at t = 3.5 s | vs. | control at t = 3.5 s | *t* | - | - |  | - | -6.687 |
|  |  |  | *p* | - | - |  | - | **< .001***** |
| experimental at t = 4 s | vs. | control at t = 4 s | *t* | - | - |  | - | -5.664 |
|  |  |  | *p* | - | - |  | - | **< .001***** |
| experimental at t = 4.5 s | vs. | control at t = 4.5 s | *t* | - | - |  | - | -4.608 |
|  |  |  | *p* | - | - |  | - | **< .01**** |
|  | | | | | | | | |
| Predictable condition (P) | | | | | | | | |
| Time in seconds | | |  | Heart rate | |  | Speed | |
|  |  |  |  | Post-cue | Post-Event |  | Post-cue | Post-Event |
| experimental at t = 0 s | vs. | control at t = 0 s | *t* | - | -0.122 |  | 0.129 | -0.565 |
|  |  |  | *p* | - | 1.000 |  | 1.000 | 1.000 |
| experimental at t = 0.5 s | vs. | control at t = 0.5 s | *t* | - | 0.261 |  | 0.182 | -1.413 |
|  |  |  | *p* | - | 1.000 |  | 1.000 | 1.000 |
| experimental at t = 1 s | vs. | control at t = 1 s | *t* | - | 0.945 |  | 0.179 | -2.705 |
|  |  |  | *p* | - | 1.000 |  | 1.000 | 1.000 |
| experimental at t = 1.5 s | vs. | control at t = 1.5 s | *t* | - | 1.756 |  | -0.772 | -4.529 |
|  |  |  | *p* | - | 1.000 |  | 1.000 | **< .01**** |
| experimental at t = 2 s | vs. | control at t = 2 s | *t* | - | 2.793 |  | -0.479 | -6.158 |
|  |  |  | *p* | - | 1.000 |  | 1.000 | **< .001***** |
| experimental at t = 2.5 s | vs. | control at t = 2.5 s | *t* | - | 3.229 |  | -1.206 | -7.710 |
|  |  |  | *p* | - | 0.457 |  | 1.000 | **< .001***** |
| experimental at t = 3 s | vs. | control at t = 3 s | *t* | - | 3.099 |  | -2.163 | -8.381 |
|  |  |  | *p* | - | 0.671 |  | 1.000 | **< .001***** |
| experimental at t = 3.5 s | vs. | control at t = 3.5 s | *t* | - | 3.310 |  | -3.129 | -8.538 |
|  |  |  | *p* | - | 0.358 |  | 0.643 | **< .001***** |
| experimental at t = 4 s | vs. | control at t = 4 s | *t* | - | 3.840 |  | -4.475 | -8.398 |
|  |  |  | *p* | - | 0.067 |  | **< .01**** | **< .001***** |
| experimental at t = 4.5 s | vs. | control at t = 4.5 s | *t* | - | 4.170 |  | -6.395 | -8.114 |
|  |  |  | *p* | - | **< .05*** |  | **< .001***** | **< .001***** |
|  | | | | | | | | |
| Predictable and Familiar condition (PF) | | | | | | | | |
| Time in seconds | | |  | Heart rate | |  | Speed | |
|  |  |  |  | Post-cue | Post-Event |  | Post-cue | Post-Event |
| t = -0.5 s | vs. | t = -0 s | *t* | 0.409 | -0.421 |  | -3.616 | -1.078 |
|  |  |  | *p* | 1.000 | 1.000 |  | **< .05*** | 1.000 |
| t = -0.5 s | vs. | t = 0.5 s | *t* | 1.063 | -0.503 |  | -6.887 | -1.625 |
|  |  |  | *p* | 1.000 | 1.000 |  | **< .001***** | 1.000 |
| t = -0.5 s | vs. | t = 1 s | *t* | 1.824 | -0.176 |  | -9.886 | -2.295 |
|  |  |  | *p* | 1.000 | 1.000 |  | **< .001***** | 1.000 |
| t = -0.5 s | vs. | t = 1.5 s | *t* | 2.856 | 0.404 |  | -12.268 | -3.132 |
|  |  |  | *p* | 0.613 | 1.000 |  | **< .001***** | 0.253 |
| t = -0.5 s | vs. | t = 2 s | *t* | 3.876 | 0.617 |  | -14.004 | -4.027 |
|  |  |  | *p* | **< .05*** | 1.000 |  | **< .001***** | **< .01**** |
| t = -0.5 s | vs. | t = 2.5 s | *t* | 4.190 | -0.027 |  | -15.114 | -4.758 |
|  |  |  | *p* | **< .01**** | 1.000 |  | **< .001***** | **< .001***** |
| t = -0.5 s | vs. | t = 3 s | *t* | 3.868 | -0.764 |  | -16.058 | -5.044 |
|  |  |  | *p* | **< .05*** | 1.000 |  | **< .001***** | **< .001***** |
| t = -0.5 s | vs. | t = 3.5 s | *t* | 3.396 | -1.072 |  | -16.610 | -5.160 |
|  |  |  | *p* | 0.102 | 1.000 |  | **< .001***** | **< .001***** |
| t = -0.5 s | vs. | t = 4 s | *t* | 3.479 | -0.936 |  | -17.233 | -5.440 |
|  |  |  | *p* | 0.076 | 1.000 |  | **< .001***** | **< .001***** |
| t = -0.5 s | vs. | t = 4.5 s | *t* | 3.671 | -1.147 |  | -17.803 | -5.771 |
|  |  |  | *p* | **< .05*** | 1.000 |  | **< .001***** | **< .001***** |
| t = -0.5 s | vs. | t = 5 s | *t* | 3.240 | -1.880 |  | -18.457 | -5.965 |
|  |  |  | *p* | 0.176 | 1.000 |  | **< .001***** | **< .001***** |
| t = -0.5 s | vs. | t = 5.5 s | *t* | 2.244 | -2.867 |  | -19.218 | -6.182 |
|  |  |  | *p* | 1.000 | 0.592 |  | **< .001***** | **< .001***** |
| t = -0.5 s | vs. | t = 6 s | *t* | 1.493 | -4.024 |  | -19.910 | -6.458 |
|  |  |  | *p* | 1.000 | **< .01**** |  | **< .001***** | **< .001***** |
| t = -0.5 s | vs. | t = 6.5 s | *t* | 1.527 | -4.381 |  | -20.054 | -6.883 |
|  |  |  | *p* | 1.000 | **< .01**** |  | **< .001***** | **< .001***** |
| t = -0.5 s | vs. | t = 7 s | *t* | 1.607 | -4.106 |  | -20.547 | -7.310 |
|  |  |  | *p* | 1.000 | **< .01**** |  | **< .001***** | **< .001***** |
| t = -0.5 s | vs. | t = 7.5 s | *t* | 1.801 | -3.344 |  | -20.825 | -7.887 |
|  |  |  | *p* | 1.000 | 0.122 |  | **< .001***** | **< .001***** |
